# ADVANCING ADOPTION AND SUSTAINABILITY OF AGE-FRIENDLY INITIATIVES THROUGH SYNERGIES AND IMPLEMENTATION SCIENCE

**DOI:** 10.1093/geroni/igad104.0333

**Published:** 2023-12-21

**Authors:** Teri Kennedy

**Affiliations:** University of Kansas Medical Center, Kansas City, Kansas, United States

## Abstract

For communities interested in adopting age-friendly principles, the plethora of age-friendly initiatives (i.e., age-, disability-, and dementia-friendly cities and communities; age-friendly universities, health systems, and public health systems) can be overwhelming. For rural, frontier, underserved, and under-resourced communities, it can be challenging to consider tackling even one such initiative. A major barrier to implementation is the know-do gap between what is known and what gets accomplished in practice. This gap is widened as a result of overly complex models and practices that are difficult to disseminate and scale, difficult to reproduce, or do not apply across settings. The know-do gap can be effectively bridged by focusing on core features common across age-friendly ecosystem initiatives. To successfully advance this work, it is important to consider strategies to maximize the collective impact of age-friendly initiatives and explore opportunities to tailor these initiatives so they can be successfully adapted for and adopted by various settings, contexts, and populations. Using an implementation science approach, three models will be introduced (Strengths-Based Interprofessional Practice and Education (SB-IPE); Micro-, Meso-, Macro- M3: Aligning Your Nexus Through a Systems Lens; and Kennedy Model of Sustainability) to facilitate the adoption and sustainability of evidence-based age-friendly principles, programs, and policies.

